# Aliskiren Prevents the Toxic Effects of Peritoneal Dialysis Fluids during Chronic Dialysis in Rats

**DOI:** 10.1371/journal.pone.0036268

**Published:** 2012-04-27

**Authors:** Juan Pérez-Martínez, Francisco C. Pérez-Martínez, Blanca Carrión, Jesús Masiá, Agustín Ortega, Esther Simarro, Syong H. Nam-Cha, Valentín Ceña

**Affiliations:** 1 Unidad Asociada Neurodeath, Departamento de Ciencias Médicas, CSIC-Universidad de Castilla-La Mancha, Albacete, Spain; 2 CIBERNED, Instituto de Salud Carlos III, Madrid, Spain; 3 Department of Nephrology, Complejo Hospitalario Universitario, Albacete, Spain; 4 Department of Research and Development, NanoDrugs, S.L., Parque Científico y Tecnológico, Albacete, Spain; 5 Department of Clinical Chemistry, Complejo Hospitalario Universitario, Albacete, Spain; 6 Department of Pathology, Complejo Hospitalario Universitario, Albacete, Spain; UAE University, Faculty of Medicine & Health Sciences, United Arab Emirates

## Abstract

The benefits of long-term peritoneal dialysis (PD) in patients with end-stage renal failure are short-lived due to structural and functional changes in the peritoneal membrane. In this report, we provide evidence for the *in vitro* and *in vivo* participation of the renin-angiotensin-aldosterone system (RAAS) in the signaling pathway leading to peritoneal fibrosis during PD. Exposure to high-glucose PD fluids (PDFs) increases damage and fibrosis markers in both isolated rat peritoneal mesothelial cells and in the peritoneum of rats after chronic dialysis. In both cases, the addition of the RAAS inhibitor aliskiren markedly improved damage and fibrosis markers, and prevented functional modifications in the peritoneal transport, as measured by the peritoneal equilibrium test. These data suggest that inhibition of the RAAS may be a novel way to improve the efficacy of PD by preventing inflammation and fibrosis following peritoneal exposure to high-glucose PDFs.

## Introduction

Chronic kidney disease is a worldwide public health problem with increasing incidence and prevalence, poor outcomes and high costs [1] Long-term peritoneal dialysis (PD) is a suitable and effective therapy option for patients with end-stage renal failure, and has been widely used for more than 20 years [2]. Nevertheless, the benefits of PD are short-lived, mainly due to structural and functional changes in the peritoneal membrane caused by the use of conventional PD fluids (PDFs) [3], [4], which contain high concentrations of glucose as the osmotic agent [5], [6]. However, a loss of peritoneal mesothelial cells (PMCs), progressive peritoneal fibrosis (PF), membrane hyperpermeability and ultrafiltration failure develop when using glucose-based solutions [7]–[9], although the physiopathological mechanisms underlying these changes are not fully understood.

Angiotensin II, a component of the renin–angiotensin–aldosterone system (RAAS), is constitutively expressed within PMCs [10], [11]. Noxious stimuli induce activation of the local peritoneal angiotensin II, which initiates production of transforming growth factor-β1 (TGF-β1), thus contributing to extracellular matrix accumulation and inducing PF [12], [13]. Functionally, these changes translate into reduced ultrafiltration capacity of the peritoneal membrane, which is a significant cause of the failure of the technique among patients on long-term PD [11]. Aliskiren decreases angiotensin II production [14] and it is therefore effective in lowering blood pressure and holds considerable potential for organ protection beyond blood pressure reduction [14], [15].

We studied the protective effects of aliskiren on PMCs exposed to glucose-enriched solutions *in vitro* as well as on the peritoneal membrane in rats dialyzed with PDFs for four weeks. We found that, at concentrations achievable in humans, aliskiren prevents high glucose-mediated PDF-induced thickening and fibrosis of the peritoneum, decreases cellular damage markers and, by decreasing fibrosis, preserves the efficacy of PDFs. These results strongly suggest that a RAAS blockade may increase the effective time of PDF therapy, paving the way for the development of new, less toxic PD solutions.

## Results

### Aliskiren Protects PMCs From PDF Toxicity *In Vitro*

**Figure 1:**
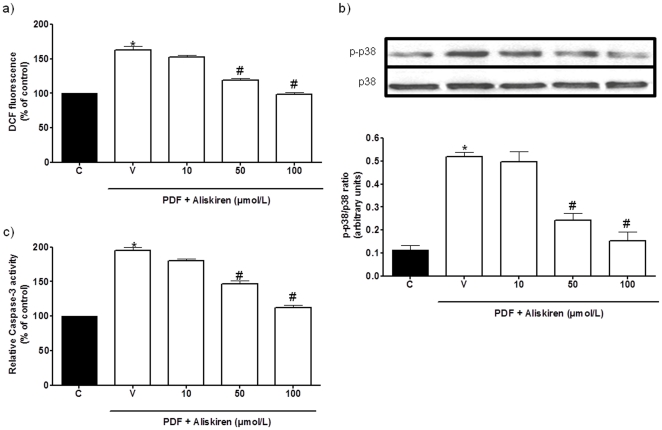
Aliskiren decreases toxicity induced by peritoneal dialysis fluids (PDFs) in rat peritoneal mesothelial cells (PMCs). a) Effect of aliskiren on PDF-mediated reactive oxygen species (ROS) production in PMCs. Cells were treated for 8 h with a 1.5%-glucose PDF diluted 1∶1 in culture medium in the absence (V) or the presence of aliskiren. ROS production was measured using dichlorodihydrofluorescein (DCF) as described in Methods. Results represent mean ± s.e.m. of 4 experiments. *p<0.05 as compared to untreated control cells (C). ^#^p<0.05 as compared to cells treated with vehicle and high-glucose PDF (V). b) Effect of aliskiren on phospho-p38 (p-p38) mitogen-activated protein kinase (MAPK)/p38 MAPK ratio in rat PMCs exposed to a high-glucose PDF for 24 h. PMCs were treated as above, in the absence (V) or presence of aliskiren and then both p-p38 MAPK and p38 MAPK protein levels were determined by western blot. The histograms represent a densitometric analysis of the p-p38 MAPK/p38 MAPK ratio. Data represent mean ± s.e.m. of 4 experiments.*p<0.05 as compared to C. #p<0.01 as compared to V. c) Effect of aliskiren on caspase-3 activity in rat PMCs exposed to a high-glucose PDF for 18 h. PMCs were treated as above, in the absence (V) or presence of aliskiren and then caspase-3 activity was determined (see Methods). Data represent mean ± s.e.m. of 4 experiments. *p<0.05 as compared to C. ^#^p<0.05 as compared to V.

**Figure 2:**
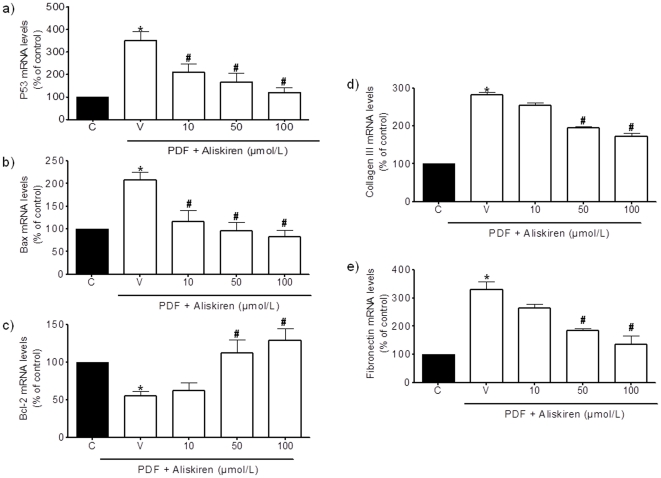
Aliskiren decreases fibrosis markers and inhibits changes in apoptosis markers induced by peritoneal dialysis fluids (PDFs) in rat peritoneal mesothelial cells (PMCs). Cells were treated for 24 h with a 1.5%-glucose PDF diluted 1∶1 in culture medium in the absence (V) or the presence of aliskiren, and the levels of mRNA for p53 (a), Bax (b), Bcl-2 (c), collagen III (d) and fibronectin (e) were determined by real-time RT-PCR. Data represent mean ± s.e.m. of 4 experiments. *p<0.05 as compared to untreated control cells (C). ^#^p<0.01 as compared to cells treated with vehicle and PDF (V).

In cultured rat PMCs, PDFs containing high levels of glucose induced increases in free-radical production that amounted to approximately 162.5±8.5% of control levels at 8 h of exposure (Fig. 1a). Phosphorylation of p38 mitogen-activated protein kinase (MAPK) is a generally accepted index of toxicity for PMCs [16]. Exposure to a high-glucose PDF for 48 h caused a marked increase in the phospho-p38 (p-p38) MAPK/p38 MAPK protein ratio compared to control cells (Fig. 1b). This toxicity may activate the cell death cascade, as indicated by the increase in caspase-3 activity observed in rat PMCs following exposure to a high-glucose PDF for 18 h (Fig. 1c). Aliskiren, at concentrations equal to or higher than 50 µmol/L, significantly prevented the observed increase in the studied toxicity markers for PMCs *in vitro* (Fig. 1). Moreover, exposure to a high-glucose PDF for 24 h caused a marked increase in mRNA levels of the pro-apoptotic markers p53 (Fig. 2a) and Bax (Fig. 2b), and a decrease in the mRNA level of the anti-apoptotic marker Bcl-2 (Fig. 2c) in rat-cultured PMCs. On the other hand, exposure to a high-glucose PDF for 24 h increased mRNA levels of fibrosis markers such as collagen III (Fig. 2d) and fibronectin (Fig. 2e). RAAS inhibition using aliskiren markedly decreased the production of these fibrosis and pro-apoptotic markers, as well and increased Bcl-2 mRNA expression (Fig. 2).

### Aliskiren Prevents Damage Induced *In Vivo* by High-Glucose PDFs

**Figure 3:**
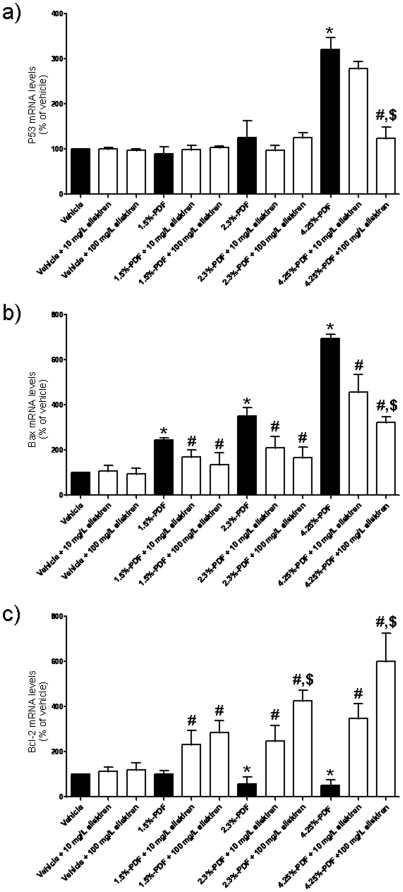
*In vivo* effect of aliskiren on p53, Bax and Bcl-2 mRNA levels in the peritoneum after daily peritoneal dialysis for 4 weeks. Twelve groups of 6 rats each one were dialyzed daily as described in Methods for 4 weeks in the absence (black histograms) and in the presence of aliskiren (10 and 100 mg/L). At the end of this period, peritoneal mesothelial cells (PMCs) were isolated and the mRNA levels for p53 (a), Bax (b), and Bcl-2 (c) quantified and normalized to the the β-actin mRNA levels. The dialysis fluid and the treatment for each group is indicated in the graph. Each histogram represents mean ± s.e.m. of 6 animals. *p<0.05 as compared to the vehicle group. ^#^p<0.05 for the aliskiren-treated groups as compared to the same PDF in absence of aliskiren. ^$^p<0.05 as compared to 10 mg/L aliskiren groups.

To explore PDF-mediated toxicity *in vivo*, we dialyzed rats daily using commercial PDFs containing three different glucose concentrations (1.5%, 2.3% and 4.5%) for four weeks. At the end of the study, the peritoneum was removed and analyzed for levels of mRNA encoding for proteins involved in the death/survival pathway. The p53 mRNA levels increased in PMCs collected from the peritoneum of rats dialyzed with 4.5% PDF as compared to the vehicle group (Fig. 3a). The addition of aliskiren (100 mg/l) to the PD solution prevented the PDF-mediated increase in p53 mRNA levels (Fig. 3a). Moreover, Bax mRNA levels were significantly higher in the rat peritoneum after chronic dialysis than in the vehicle-dialyzed group. The increase in Bax mRNA levels following dialysis was much smaller when aliskiren was added to the PDF (Fig. 3b), with a concentration-dependent effect from aliskiren. The above data indicate that aliskiren prevents the PDF-induced increase in pro-apoptotic gene expression. In addition, chronic dialysis using 2.3% and 4.5% PDFs decreased mRNA levels of the anti-apoptotic protein Bcl-2 in the peritoneum (Fig. 3c). The addition of aliskiren to the PD solution markedly increased Bcl-2 mRNA levels well above basal levels (between 2- and 5-fold) (Fig. 3c). Surprisingly, this effect was not observed in vehicle-dialyzed rats and was only evident following chronic dialysis with PDF, suggesting that dialysis with bio-incompatible PD solutions may sensitize peritoneal cells to the actions of aliskiren.

### Aliskiren Reduces Inflammation and Fibrosis Produced by High-Glucose PDFs

**Figure 4:**
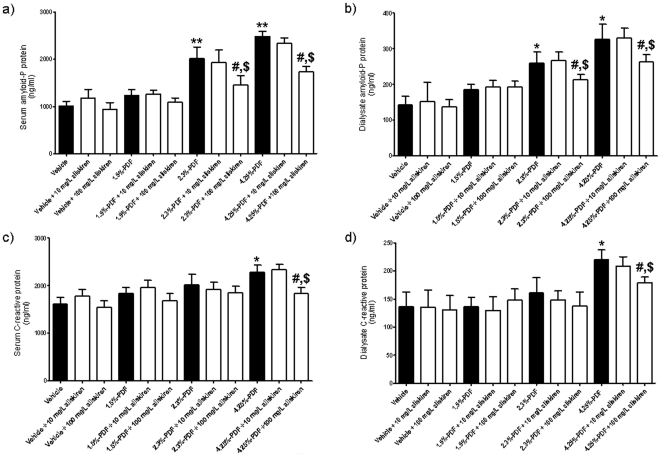
Aliskiren decreases the levels of inflammatory markers *in vivo* in both serum and dialysate after chronic peritoneal dialysis. The groups of animals were the same as in Fig. 3. A Peritoneal Equilibration Test (PET) was performed for 2 h at the end of the 4 weeks of dialysis. The levels of amyloid-P protein (a, b) and C-reactive protein (c, d) were determined in both serum (a, c) and dialysate (b, d) collected after 2 h dwell time. The dialysis fluid used and the treatment for each group is indicated in the graph. Each histogram represents mean ± s.e.m. of 6 animals. *p<0.05 as compared to the vehicle group. **p<0.01 as compared to the vehicle group. ^#^p<0.05 for the aliskiren-treated groups as compared to the same PDF in absence of aliskiren. ^$^p<0.05 as compared to 10 mg/L aliskiren groups.

After four weeks of daily PD, a Peritoneal Equilibrium Test (PET) adjusted for rats was performed using 2.3% PDF. The C reactive protein (CRP) and amyloid-P protein level inflammation markers were increased in both serum and dialysate when the rats were dialyzed using 4.25% PDF in the absence of aliskiren (Figs. 4a and 4b). Moreover, amyloid-P protein levels were also significantly increased in the group dialyzed with 2.3% PDF. The addition of aliskiren (100 mg/l) to the PDFs prevented the increase observed in the levels of inflammation markers CRP and amyloid-P protein in both serum and dialysate (Figs. 4c and 4d).

**Figure 5:**
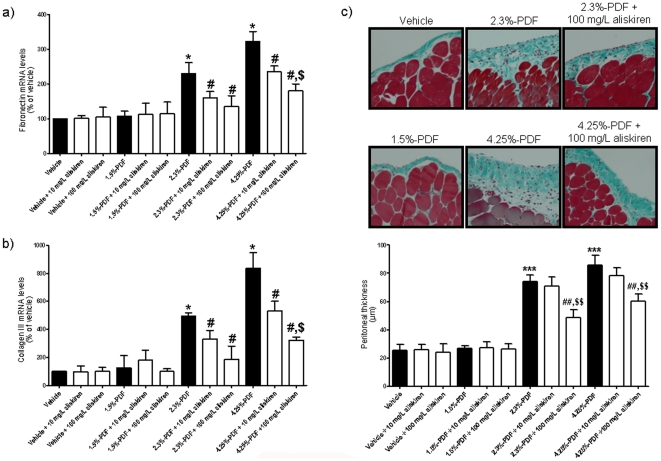
Aliskiren reduces the peritoneal fibrosis *in vivo* after chronic peritoneal dialysis. The groups of animals were the same as in Fig. 3. At the end of the 4 week-dialysis period, peritoneal mesothelial cells (PMCs) were isolated and the fibronectin (a) and collagen III (b) mRNA levels were determined in PMCs and normalized to the β-actin mRNA levels. The dialysis fluid used and the treatment for each group is indicated in the graph. Each histogram represents mean ± s.e.m. of 6 animals. *p<0.05 as compared to the vehicle group. ^#^p<0.05 for the aliskiren-treated groups as compared to the same PDF in absence of aliskiren. ^$^p<0.05 as compared to 10 mg/L aliskiren groups. c) Masson's trichrome staining of parietal peritoneum. Top panel. Histological sections from the vehicle, 1.5%-PDF, 2.3%-PDF, 4.25%-PDF, 2.3%-PDF plus aliskiren (100 mg/L) and 4.25%-PDF plus aliskiren (100 mg/L) groups at 200× magnification. Bottom panel. Quantification of the peritoneal thickness. The dialysis fluid used and the treatment for each group is indicated in the graph. Each histogram represents mean ± s.e.m. of 6 animals. ***p<0.001 as compared to the vehicle group. ^##^p<0.01 for the aliskiren-treated groups as compared to the same PDF in absence of aliskiren. ^$$^p<0.01 as compared to 10 mg/L aliskiren groups.

Consistent with well-known PD complications, fibrosis markers such as fibronectin and collagen III mRNA levels were markedly elevated in PMCs collected from the 2.3% and 4.25% PDF groups when compared to the vehicle group (Figs. 5a and 5b). These changes in fibronectin and collagen III gene expression after PDF exposure correlated with an increase in the thickness of the peritoneal membrane (Fig. 5c). The inclusion of aliskiren in the PD solution markedly reduced fibronectin and collagen III mRNA levels in response to chronic dialysis with the 2.3% and 4.25% PDFs and decreased the thickness of the peritoneal membrane after chronic dialysis (Fig. 5).

### Aliskiren Inhibits High-Glucose PDF-Mediated Changes in Peritoneal Solute Transport

**Figure 6:**
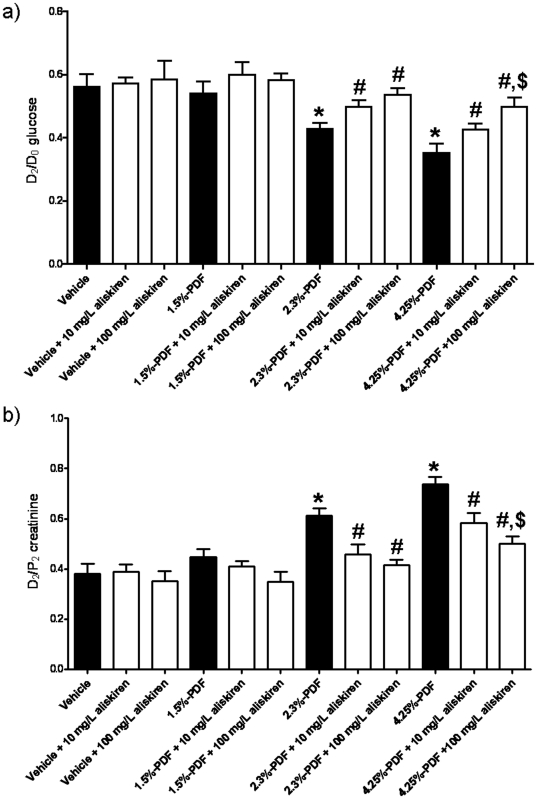
Aliskiren reduces changes in solute transport through the peritoneal membrane during chronic dialysis using high-glucose peritoneal dialysis fluids. The groups of animals were the same as in Fig. 3. A Peritoneal Equilibration Test (PET) was performed for 2 hours at the end of the 4 weeks of dialysis and the ratios D_2_/D_0_ glucose level (a) and D_2_/P_2_ creatinine level (b) were determined. The dialysis fluid used and the treatment for each group is indicated in the graph. Each histogram represents mean ± s.e.m. of 6 animals. *p<0.05 as compared to the vehicle group. ^#^p<0.05 for the aliskiren-treated groups as compared to the same PDF in absence of aliskiren. ^$^p<0.05 as compared to 10 mg/L aliskiren groups.

There was no significant difference in D_2_/D_0_ glucose and D_2_/P_2_ creatinine ratios between the groups dialyzed with vehicle in the absence or presence of aliskiren (Fig. 6). The peritoneal solute transport in the group dialyzed with 1.5% PDF for four weeks was no different from that observed in the group dialyzed with saline alone (vehicle group), and the presence of different doses of aliskiren in the 1.5% PDF did not have any effect. On the other hand, D_2_/D_0_ glucose ratios were significantly lower, and D_2_/P_2_ creatinine ratios were significantly higher in the 2.3% and the 4.25% PDF groups as compared to the vehicle group (Fig. 6) indicating a high transporter status that is consistent with the observed inflammatory changes. The groups dialyzed with both 2.3% and 4.25% PDFs supplemented with aliskiren showed higher D_2_/D_0_ glucose and lower D_2_/P_2_ creatinine ratios than their respective groups dialyzed with high-glucose PDFs in the absence of aliskiren indicating a lower transporter status. Moreover, the effect of aliskiren was dose-dependent, with a significantly higher D_2_/D_0_ glucose ratio and a significantly lower D_2_/P_2_ creatinine ratio in the group dialyzed with 4.25% PDF supplemented with 100 mg/l aliskiren than the group dialyzed with 4.25% PDF supplemented with 10 mg/l aliskiren (Fig. 6).

## Discussion

PD is an effective therapy for patients with end-stage renal failure [8]. Nevertheless, the benefits of PD are short-lived, due mainly to structural and functional changes in the peritoneal membrane caused by the use of conventional high-glucose PDFs. These changes can cause deterioration of the peritoneal membrane, thereby inducing a failure in peritoneal transport of solutes and ultrafiltration [17]. This failure results in patients having to turn to hemodialysis therapy for end-stage renal failure, and enduring the medical, social and economic limitations related to this type of treatment.

There is substantial evidence to support a pathogenic role of high-glucose PDFs in the development of structural and functional alterations in the peritoneum of long-term PD patients, including an increase in oxidative stress, p38 MAPK activity and apoptosis [18], [19]. Previous studies have shown that all these PMC damage markers are increased in PMCs exposed to high-glucose solutions in a dose- and time-dependent manner [19], [20]. We observed in our study that rat PMCs exposed *in vitro* to a high-glucose PDF showed increases in damage markers, such as reactive oxygen species (ROS) production, p38 MAPK phosphorylation, caspase-3 activity and mRNA expression of p53 and Bax. The addition of aliskiren to the culture medium markedly reduced damage to PMCs in culture suggesting that a blockade of angiotensin synthesis may be beneficial during PD by protecting PMCs from high-glucose PDF-mediated damage. These protective actions of aliskiren may be explained by the known growth factor properties of angiotensin II, which when over-produced may cause PF [12]. Moreover, angiotensin receptor blockers and angiotensin converting-enzyme inhibitors ameliorate chlorhexidine gluconate-induced PF in rats [21]. On the other hand, high-glucose-induced angiotensin II synthesis has been observed in cardiac fibroblasts [22], vascular smooth muscle cells [23] and renal mesangial cells [24], among others. Furthermore, angiotensin II produced by PMCs mediates high-glucose PDF-induced up-regulation of TGF-β1 and fibronectin expression and this up-regulation is mediated by ROS [10]. Aliskiren inhibits angiotensin I generation from angiotensinogen by inhibiting renin leading to decreased angiotensin II production [14]. *In vitro* studies have shown that local RAAS is physiologically active in many cell types, including PMCs. An RAAS inhibitor could therefore be a powerful tool for preserving peritoneal function during PD.

As with our results, previous studies have shown that the deleterious effect of chronic PD using PDFs with elevated glucose levels is characterized by the activation of apoptotic processes, accumulation and deposition of excess matrix proteins within the interstitial area, neoangiogenesis and vasculopathy of the peritoneal microvasculature [25], [26]. In our study, we analyzed the effect of aliskiren addition to PD solutions during chronic (28 days) dialysis of rats. We found that PMCs isolated from the peritoneum of rats dialyzed daily for four weeks showed increased mRNA levels of fibrosis (collagen III and fibronectin) and pro-apoptotic (p53 and BAX) markers, as well as decreased Bcl-2 mRNA (an anti-apoptotic marker), similar to that observed in acutely isolated cultured PMCs *in vitro,* indicating that the response to high-glucose PDFs is similar *in vitro* and *in vivo*. This expression pattern suggests that, during chronic PD, PMCs suffer damage that activates death-signaling pathways. However, in rats treated with aliskiren-containing PDFs, changes in those cell damage markers were significantly reduced, which is consistent with recent studies showing that aliskiren suppressed *in vivo* gene expression of pro-apoptotic factors inhibiting degeneration [27], [28]. Taken as a whole, these data indicate that aliskiren may protect PMCs *in vivo*, which would be useful to extending the period during which PD may be used without peritoneal damage and thus delaying the initiation of hemodialysis.

After peritoneal membrane injury (especially to the mesothelial cell layer), a process of tissue repair begins. This process can be described as an inflammatory response that is characterized by remesothelialization of the wounded area, neovascularization and fibrosis of the submesothelial cell extracellular matrix [29]. We observed that levels of fibrosis markers fibronectin and collagen III mRNAs were significantly lower in PMCs collected from animals dialyzed with PDFs containing aliskiren than in control animals dialyzed with PDFs lacking aliskiren. Accordingly, high-glucose PDFs also increased the thickness of the peritoneum, as well as serum and dialysate CRP (one of the acute phase proteins whose levels increase during systemic inflammation) and amyloid-P protein (an acute phase protein during inflammation that can bind to apoptotic and necrotic cells) levels. All these changes were at least partially reversed by the addition of aliskiren to the PDFs. This result supports the idea that aliskiren protects PMCs from PDF-induced damage, thus decreasing the inflammation and fibrosis associated with peritoneal PMC damage.

One important issue is whether these protective actions of aliskiren are correlated with improved peritoneal membrane function during PD. To explore this issue, we performed a PET, which reflects the rates of glucose and creatinine transfer through the peritoneal membrane, after four weeks of PD using high-glucose PD solutions. Chronic dialysis using high-glucose PDFs produced a glucose-dependent decrease in the D_2_/D_0_ glucose ratio and an increase in the creatinine D_2_/P_2_ ratio, indicating a high transporter status. This type of transport is generally associated with less efficient ultrafiltration due to the more rapid absorption of glucose and an earlier loss of the osmotic driving force for fluid transport across the peritoneal membrane [5], which leads to an early transfer to hemodialysis due to failure of the PD [30]. In addition, a high transporter status is also associated with reduced survival of patients receiving PD [31]. The addition of aliskiren to the PDFs during chronic dialysis markedly increased the D_2_/D_0_ glucose ratio while decreasing the creatinine D_2_/P_2_ ratio, thus slowing the transport rate to values obtained in rats dialyzed with vehicle, which was due to the protective action on PMCs against PDF-induced damage. This indicates that the addition of aliskiren markedly improves the efficacy of PD, probably by preventing PMC damage and subsequent inflammation and fibrosis following peritoneal exposure to high-glucose PDFs.

We therefore propose that inhibition of the RAAS by aliskiren protects against high-glucose PDF-induced oxidative stress, apoptosis, inflammation, and fibrosis *in vitro* and *in vivo*. Moreover, the addition of aliskiren to PDFs prevents alterations in peritoneal transport as measured by the PET. These data suggest that inhibition of the RAAS may be a novel solution for preventing long-term PD-related modifications in the peritoneal membrane, thereby improving the efficacy of PD.

## Materials and Methods

### Materials

All chemicals, unless otherwise stated, were obtained from Sigma-Aldrich Chemical Company (St. Louis, MO, USA), and all tissue culture plastics were purchased from TPP (Trasadingen, Switzerland).

### Animals

Seventy-two female Sprague–Dawley rats weighing 200 g to 220 g (Charles River Breeding Laboratories) were used for *in vivo* experiments. The animals were housed at a constant room temperature, with 12-hour light and dark cycles. Food and water were given *ad libitum*. All experimental protocols were carried out in accordance with the European Community Council Directive 2003/65/CE and with the experimental protocols approved by the CHU Albacete Institutional Animal Care and Use Committee.

### Dialysis Experimental Design

Rats were randomly divided into 12 groups of 6 animals each. Four different dialysates were used: Vehicle (saline), 1.5% glucose PDF (1.5% PDF; CAPD/DPCA 2, Fresenius Medical Care, St. Wendel, Germany), 2.3% glucose PDF (2.3% PDF; CAPD/DPCA 4, Fresenius Medical Care) and 4.25% glucose PDF (4.25% PDF; CAPD/DPCA 3, Fresenius Medical Care). Each dialysate was administrated to three different groups incorporating aliskiren at 0 mg/l, 10 mg/l (low-dose) or 100 mg/l (high-dose) in the dialysate. The animals received 20 ml dialysate via a 30-gauge needle daily for four weeks. Furthermore, a group of animals was not dialyzed and the data obtained were not significantly different from animals dialyzed with vehicle (data not shown). Body weight was monitored at the beginning and the end of the experimental period. No rats were lost and all animals appeared to be healthy during the study, which involved repeated infusions of PDF.

### Peritoneal Equilibration Test (PET)

After four weeks of treatment with the dialysates, a PET was performed during a 2-hour dwell with a 2.3% glucose PD solution. PETs were also performed on rats from the control group. During each PET, rats were given 30 ml of conventional 2.3% PDF (Fresenius Medical Care) via a 30-gauge needle to the peritoneal cavity. Dialysate samples (1 ml) were taken at time 0 (immediately after infusion) and 120 minutes after infusion. Blood samples were taken from the tail vein at the start and end of the PET. During the PET, animals were awake and had free access to water and food.

The dialysate and blood samples were centrifuged and stored at −20°C until assayed. Concentrations of creatinine and glucose in serum and dialysate were measured using enzymatic methods on a DSX automated analyzer (DYNEX Technologies Inc., Chantilly, VA, USA). Amyloid-P protein and CRP quantification in serum and dialysate were measured using ELISA kits (GenWay Biotech Inc., San Diego, CA, USA). Peritoneal solute transport was calculated from the dialysate concentration at 2 h relative to its concentration in the initial infused dialysis solution (D_2_/D_0_ glucose) for glucose, and the dialysate-to-plasma concentration ratio (D_2_/P_2_ creatinine) at 2 h for creatinine.

At the end of the PET, animals were euthanized and biopsies of the parietal peritoneum were taken for light microscopy. Rat PMCs were also isolated from the parietal peritoneum and cultured as described below. Similar samples were taken from rats not exposed to PD solution. Peritoneal thickness was determined in Masson's trichrome stained tissue specimens by measurement of the combined thickness of the mesothelium and submesothelial interstitium using a 200× objective lens via a computer imaging analysis system (Image-Pro Plus, Media Cybernetics, Bethesda, MD, USA) as described previously [32]. The thickness of the parietal peritoneum was measured at a minimum of 10 points in each sample.

### Rat Peritoneal Mesothelial Cells (PMCs) Culture

Rat PMCs were isolated from the peritoneum of rats by enzymatic digestion as previously described [33]. To identify PMCs, the cells were examined for specific markers and morphology as described elsewhere [33]. Cells between passages 3 and 6 were used for experiments. Experimental procedures were carried out according to the guidelines of the European Community on Welfare of Research Animals (Directive 2003/65/CE) and the CHU Albacete Institutional Animal Care and Use Committee.

To determine the effect of high-glucose solutions, subconfluent PMCs were incubated with serum-free media for 24 h to arrest and synchronize cell growth as previously described [34]. The PMCs were then treated for 48 h with serum-free media containing aliskiren (10 µmol/l to 100 µmol/l) or vehicle (distilled water), before stimulation with a 1.5% glucose PDF diluted to twice the volume with serum-free M199 medium in the presence or absence of aliskiren.

### Reactive Oxygen Species (ROS) Determination

Intracellular formation of ROS was detected using 5-(and-6)-chloromethyl-2,7-dichlorodihydrofluorescein diacetate (DCF [CM-H2DCFDA: Invitrogen, Barcelona, Spain]), as previously described [35]. DCF was added at a final concentration of 10 µmol/l, and the cells were incubated for 60 minutes at 37°C in the dark. Cells were then washed, resuspended in PBS, and kept on ice for immediate detection by flow cytometry (BD-LSR, BD Biosciences, San Jose, CA, USA). Data were acquired and analyzed using the CellQuest program (BD Biosciences).

### Western Blot Analysis

Proteins (20 µg/lane) were separated on 10% polyacrylamide-SDS gels in reducing conditions [36]. After electrophoresis, samples were transferred onto nitrocellulose membranes (Bio-Rad Laboratories, Hercules, CA, USA), blocked with 5% nonfat milk in blocking TTBS buffer (50 mmol/l Tris HCl, 150 mmol/l NaCl, and 0.05% Tween 20; pH 7.5), and incubated overnight at 4°C with a mouse monoclonal anti-p-p38 MAPK antibody (1∶1.000, Cell Signaling, Beverly, MA, USA) or a rabbit polyclonal anti-p38 MAPK antibody (1∶1000, Santa Cruz Biotechnology, Santa Cruz, CA, USA). Afterwards, blots were washed and incubated at room temperature for 1 h with a secondary antibody and developed using an enhanced chemiluminescence system (Millipore, Bedford, MA, USA). Densitometric analysis of immunoreactive bands was performed using Quantity One Software (Bio-Rad Laboratories).

### Caspase-3 Activity

Caspase-3 activity was determined as previously described [37]. Cell extracts (40 µg of protein) were incubated in reaction buffer (25 mmol/l HEPES, 10% sucrose, 0.1% CHAPS buffer, and 10 mmol/l DTT) containing 50 µmol/l fluorescence substrate Asp-Glu-Val-Asp-7-amino-4 trifluoromethyl-coumaryl (Z-DEVD-AFC) at 37°C for 1 h. Cleavage of the AFC fluorophore was determined at 37°C on an Infinite 200 microplate reader (Tecan Group, Salzburg, Austria) at an excitation wavelength of 400 nm and a fluorescence emission wavelength of 505 nm.

### Real-time RT-PCR Analysis

RNA expression was evaluated by real-time RT-PCR in cultured PMCs isolated from rats used for *in vivo* experiments. cDNA was synthesized from the purified total RNA using a High Capacity cDNA Reverse Transcription Kit (Applied Biosystems, Foster City, CA, USA) according to the manufacturer's instructions. For real-time RT-PCR, cDNA was amplified using SYBR Green PCR Master mix with the StepOne Real-Time PCR System and StepOne v2.0 software (Applied Biosystems). The following primer sets were used to amplify: fibronectin, 5′-GCA-CAG-GGG-AAG-AAA-AGG-AG-3′ (sense) and 5′-TTG-AGT-GGA-TGG-GAG-GAG-AG-3′ (antisense); collagen III, 5′-ATA-TCA-AAC-ACG-CAA-GGC-3′ (sense) and 5′-GAT-TAA-AGC-AAG-AGG-AAC-AC-3′ (antisense); p53, 5′-CCT-CCT-CAG-CAT-CTT-ATC-CG-3′ (sense) and 5′-CAC-AAA-CAC-GCA-CCT-CAA-A-3′ (antisense); Bax, 5′-GAT-GCG-TCC-ACC-AAG-AA-3′ (sense) and 5′-AGT-AGA-AGA-GGG-CAA-CCA-C-3′ (antisense); and Bcl-2, 5′-CCC-AAG-GGA-AGA-CGA-TG-3′ (sense) and 5′-GAG-CGG-GTA-GGG-AAA-GA-3′(antisense). The real-time RT-PCR reaction was maintained at 95°C for 10 minutes, followed by 40 cycles of 95°C for 15 seconds and 60°C for 1 minute. The dissociation curves were analyzed to ensure amplification of a single PCR product. In order to ensure reliability of the results, all samples were processed in triplicate. Quantification was performed by the comparative cycle threshold (Ct) method [38]. To normalize the data, the β-actin RNA expression level was used as a housekeeping gene.

### Statistical Analysis

Statistical significance was evaluated by nonparametric variance analysis (Kruskal-Wallis) followed by Dunn's test, with *p*<0.05 considered statistically significant. The SPSS software application (version 13.0: SPSS, Chicago, IL) was used for all statistical analyses.
